# 1018. Case Study. Cannabinoid Hyperemesis, Burn Injury and Nutritional Support: A Case Report and Clinical Implications

**DOI:** 10.1093/jbcr/irag033.286

**Published:** 2026-04-06

**Authors:** Christopher T Borchers, Michael J Feldman

**Affiliations:** Virginia Commonwealth University, Richmond, VA; Virginia Commonwealth University, Richmond, VA

## Abstract

**Patient Presentation (age range, injury details, relevant history):**

A 20–29 year-old female with a history of major depressive disorder, generalized anxiety disorder, cannabinoid hyperemesis syndrome (CHS), and peptic ulcer disease was found unresponsive in the shower with 20% TBSA mixed partial and full-thickness scald burns to the posterior torso. She was admitted to the burn unit for resuscitation and wound care. Family reported recurrent hot water exposure as a maladaptive strategy to manage CHS symptoms, contributing to prior scald injuries.

**Clinical Challenges:**

CHS presents diagnostic challenges and can mimic acute medical emergencies, with complications including dehydration, acute kidney injury (AKI), and rhabdomyolysis. This patient’s course was complicated by AKI requiring continuous renal replacement therapy, recurrent hyperemesis, and poor nutritional intake.

**Management Approach:**

Enteral nutrition was initiated early via post-pyloric feeding tube (PPFT) on hospital day (HD) 2 but was discontinued after the tube was vomited out on HD5 and not replaced due to patient intolerance. On HD3, 5, and 7, she experienced repeated bouts of emesis with associated nutritional decline. Nearing discharge, HD23 and 24, the patient again experienced episodes of hyperemesis, believed to be self-induced.

Medical management included scheduled dronabinol and PRN ondansetron, prochlorperazine, and haloperidol.

Operative burn care included excision and allograft placement on HD4, followed by autograft and autologous spray keratinocyte suspension on HD10. Due to poor nutritional tolerance, total parenteral nutrition (TPN) was initiated on HD10 and continued until HD20.

**Outcomes:**

Dressing takedown on HD13 showed well-adhered grafts with some maceration and partial loss. Nutritional markers remained stable (prealbumin: 14 on HD8 and HD15, 15 on HD21).

Microbiological studies revealed *E. coli* UTI and MRSA colonization on admission; no hospital-acquired infections occurred.

HD25, the patient was discharged home with family support, wounds healed, and transitioned to open air care with topical moisturizers.

**Lessons Learned:**

This case highlights the intersection of psychiatric illness, CHS-related behaviors, burn injury, and nutritional challenges. CHS, linked to chronic cannabis use and compulsive hot bathing, requires early recognition and aggressive multidisciplinary management. Optimal outcomes depended on coordinated care from burn surgery, nephrology, psychiatry and nutrition.

**Applicability to Practice:**

CHS is a challenging condition that can significantly impact clinical outcomes. Improved recognition of CHS risk factors, symptoms, and diagnostic criteria may help reduce unnecessary investigations and resource utilization. Nutritional support remains critical in wound management and healing; while total parenteral nutrition (TPN) is a viable option, it carries inherent risks and must be carefully managed.

Optimal outcomes depend on the expertise of a multidisciplinary team, including surgical, medical, nutritional, and psychiatric specialists. This case highlights the importance of thorough review of patient history, as underestimating high-risk behaviors can have serious clinical consequences.

